# Prior X-Ray and Diagnostic Yield of Knee MRI: A Retrospective Study of Imaging Pathways and Healthcare Utilization

**DOI:** 10.3390/healthcare14121628

**Published:** 2026-06-09

**Authors:** Bandar Alwadani

**Affiliations:** Department of Diagnostic Radiography Technology, College of Nursing and Health Sciences, Jazan University, Jazan 45142, Saudi Arabia; balwadani@jazanu.edu.sa

**Keywords:** knee MRI, diagnostic yield, healthcare utilization, referral patterns, imaging stewardship

## Abstract

Purpose: Variation in knee MRI diagnostic yield is often interpreted as reflecting imaging effectiveness. However, in real-world healthcare systems, diagnostic yield may instead be driven by referral behavior and patient selection. Understanding this distinction is essential for evaluating imaging utilization, healthcare efficiency, and potential overuse of advanced imaging. This study examines whether differences in MRI yield reflect imaging pathways or underlying referral patterns in routine clinical practice. Materials and Methods: This retrospective cohort study included consecutive patients undergoing knee MRI between January 2020 and December 2024. Patients with red flag indications were excluded to focus on discretionary imaging. The primary outcome was clinically relevant MRI findings based on final report impressions. The primary exposure was prior X-ray before MRI. Multivariable logistic regression was used for adjusted analysis, including age, sex, trauma status, mechanical symptoms, and symptom duration. Results: Among 486 patients, 59.5% had prior X-ray. Clinically relevant MRI findings were less frequent among patients with prior X-ray (40.1%) than among those without (49.7%), corresponding to an absolute difference of 9.6%. After adjustment for sex and clinical covariates, prior X-ray showed lower odds of clinically relevant findings, although this association was attenuated and no longer statistically significant (aOR 0.74, 95% CI 0.50–1.10; *p* = 0.138). Male sex was independently associated with higher odds of clinically relevant MRI findings (aOR 2.48, 95% CI 1.61–3.83; *p* < 0.001). Formal interaction testing did not demonstrate significant effect modification by trauma status (*p* = 0.317). These findings suggest that variation in MRI yield may reflect differences in referral pathways, patient selection, and healthcare utilization patterns. Conclusions: MRI yield in routine practice may be influenced by differences in clinical context and referral-related patient selection. Further studies are needed to better understand the contribution of imaging pathways to observed variation in diagnostic yield.

## 1. Introduction

Variation in knee magnetic resonance imaging (MRI) diagnostic yield is commonly interpreted as reflecting imaging effectiveness in clinical practice. However, this assumption may be misleading, as observed MRI yield may reflect not only imaging performance but also how patients are selected for imaging.

MRI plays a central role in the evaluation of knee disorders, offering high sensitivity for detecting intra-articular and soft tissue abnormalities [1]. Its use has increased substantially in routine practice, raising concerns regarding appropriateness and efficiency, particularly when performed early in the diagnostic pathway without prior basic imaging [2].

Plain X-ray remains the most accessible and widely recommended initial imaging modality for knee assessment, especially in non-acute and degenerative presentations [3,4]. It can identify structural abnormalities that explain symptoms and may reduce the need for further imaging. Despite this, MRI is frequently performed without prior X-ray in real-world clinical settings, reflecting variation in referral patterns and clinical decision-making [2].

This variation in imaging pathways has important clinical and system-level implications. Low-yield MRI examinations may contribute to increased healthcare costs, prolonged waiting times, and reduced efficiency of imaging services [5]. Implicitly, variation in MRI yield is often attributed to imaging sequence. However, this assumption has not been directly evaluated in routine clinical practice.

Several studies have evaluated different aspects of knee MRI utilization and appropriateness. Gómez-García et al. reported that a substantial proportion of knee MRI examinations requested in primary care were not fully aligned with appropriateness recommendations, highlighting opportunities to improve imaging stewardship and referral practices [6]. Similarly, George et al. demonstrated that knee MRI is frequently performed without prior radiography despite established imaging recommendations, suggesting variability in imaging pathways and potential inefficiencies in healthcare utilization [7]. In contrast, much of the broader MRI literature has focused on diagnostic accuracy and disease detection rather than healthcare utilization pathways or referral-related determinants of diagnostic yield [8]. Collectively, these studies highlight important issues related to MRI appropriateness, utilization, and diagnostic performance; however, the extent to which variation in knee MRI diagnostic yield is associated with differences in imaging pathways and referral-related patient selection remains insufficiently explored. This distinction is important because differences in yield may reflect variation in who undergoes MRI in addition to differences in imaging performance itself.

From a healthcare system perspective, distinguishing between imaging effectiveness and referral-driven variation in diagnostic yield is critical. Misinterpreting diagnostic yield as a direct indicator of imaging performance may lead to inaccurate conclusions regarding healthcare utilization, resource allocation, and potential overuse of advanced imaging. Therefore, this study aimed to evaluate the association between prior X-ray and the likelihood of clinically relevant MRI findings in a real-world clinical cohort. By focusing on routine practice, this study seeks to clarify whether observed variation in MRI yield is more closely related to imaging sequence or to patient selection and to provide a clinically relevant perspective for interpreting MRI utilization in radiology practice.

## 2. Materials and Methods

### 2.1. Study Design and Setting

This retrospective observational study included all consecutive knee magnetic resonance imaging (MRI) examinations performed over a five-year period (January 2020 to December 2024) at a single tertiary care institution.

Data were obtained from the radiology information system (RIS) and electronic medical records (EMRs), reflecting routine clinical practice. Imaging protocols and reporting standards remained stable throughout the study period. MRI examinations were performed using both 1.5-T and 3-T systems under standardized institutional workflows.

The study was designed to evaluate imaging pathways as they occur in routine clinical practice, rather than under controlled or idealized diagnostic conditions, with a specific focus on the association between prior X-ray and the diagnostic yield of MRI.

This study was conducted in accordance with the Strengthening the Reporting of Observational Studies in Epidemiology (STROBE) guidelines [9].

### 2.2. Study Population

All consecutive patients undergoing knee MRI during the study period were eligible.

Patients with red flag indications were excluded. Red flags were defined using a predefined binary variable recorded at the time of referral and included suspected serious pathology such as infection, malignancy, or acute fracture. These cases were excluded because MRI use in such scenarios is non-discretionary and does not reflect routine imaging decision-making. Trauma status was defined independently of red-flag indications and referred to a documented history of knee injury recorded in the referral information. Therefore, patients presenting with traumatic symptoms or suspected soft-tissue injuries, including ligamentous, meniscal, or bone contusion injuries, remained eligible for inclusion provided that acute fracture was not suspected at the time of referral.

After exclusion of red flag cases (n = 8), the final analytic cohort consisted of 486 patients.

### 2.3. Exposure Variable

The primary exposure was prior X-ray, defined as a documented radiographic examination of the same knee performed before MRI within the same episode of care.

The episode of care was defined as within 3 months prior to MRI, a pragmatic interval reflecting routine referral patterns, selected to balance temporal relevance with completeness of prior imaging capture. Patients who underwent MRI after prior radiography had excluded acute fracture were not excluded from the study. Such cases remained eligible for inclusion and were classified according to the predefined exposure definition based solely on the presence or absence of prior ipsilateral knee radiography within the specified 3-month exposure window.

Laterality was verified to ensure that the X-ray and MRI corresponded to the same knee.

Only X-ray examinations performed prior to MRI were considered, ensuring correct temporal ordering of exposure and outcome.

This variable was extracted directly from RIS data and coded as a binary variable (yes/no). No assumptions were made regarding the indication for or findings of the prior X-ray.

The study focused on the presence or absence of prior radiographic utilization rather than the diagnostic content of radiographic findings. Radiographic reports were not consistently available in a structured format suitable for reliable retrospective extraction across the entire study period and therefore were not incorporated into the analysis.

### 2.4. Outcome Definition

The primary outcome was the presence of clinically relevant MRI findings. Clinically relevant findings were defined as abnormalities explicitly documented in the final impression section of the MRI report and interpreted by the reporting radiologist as explanatory of the presenting complaint or likely to influence clinical management. This definition reflects findings most relevant to clinical decision-making and serves as a pragmatic proxy for clinically actionable outcomes in the absence of standardized outcome linkage, such as surgical confirmation or treatment change. Although not linked to downstream clinical outcomes, this approach captures the decision-relevant information used in real-world clinical workflows. This definition aligns with how imaging findings are operationalized in routine clinical decision-making.

This approach reflects the operational definition of clinically meaningful findings as used in routine radiology reporting, rather than an externally adjudicated reference standard. To enhance reproducibility and reduce subjective interpretation, outcome classification was restricted strictly to the final impression section, avoiding extraction from descriptive report text. This approach prioritizes clinical interpretability over purely structural classification, aligning the outcome definition with real-world decision-making processes.

No secondary reinterpretation was performed. While formal interobserver validation was not conducted, this design minimizes investigator-driven classification bias and preserves ecological validity.

### 2.5. Secondary Outcome

A secondary outcome was defined as low-yield MRI examinations (absence of clinically relevant findings), used as a proxy for potentially non-actionable or low-value imaging in routine healthcare practice.

### 2.6. Covariates

The following prespecified variables were included based on clinical relevance:

- Sex (binary; male/female)
- Age (continuous, in years for interpretability and parsimony)
- Trauma status (binary; acute injury versus non-traumatic presentation as documented in the referral)
- Mechanical symptoms (binary; defined as locking, catching, or instability explicitly documented in the clinical referral or EMR. Only clearly documented symptoms were coded as present; missing or non-specific descriptions were coded as absent)
- Symptom duration (binary; categorized as acute (≤6 weeks) or chronic (>6 weeks), consistent with commonly used clinical thresholds)

All covariates were included in the multivariable model without data-driven selection.

### 2.7. Data Extraction

Data were extracted from structured RIS and EMR fields using predefined queries. No manual reinterpretation of imaging findings was performed. All variables reflect routine clinical documentation. While this may introduce variability related to documentation quality, it enhances external applicability to comparable real-world clinical environments.

Documentation of clinical variables depended on referral quality and may be subject to variability. This variability reflects real-world clinical documentation rather than controlled research conditions. Detailed indication granularity beyond available structured variables was not consistently captured. While conducted in a single-center setting, the reliance on routine clinical data enhances external applicability to similar real-world imaging environments.

### 2.8. Prespecified Sensitivity Analysis

A prespecified sensitivity analysis excluding patients with documented osteoarthritis was conducted to evaluate the robustness of the association in a subgroup less likely to have chronic degenerative findings.

### 2.9. Statistical Analysis

Continuous variables are presented as mean ± standard deviation, and categorical variables as counts and percentages. Logistic regression models were used to evaluate the association between prior X-ray and clinically relevant MRI findings.

Baseline descriptive comparisons between groups were supplemented with standardized mean differences to assess the magnitude of baseline imbalance.

Formal interaction testing between prior X-ray and trauma status was performed to evaluate potential effect modification while preserving model interpretability.

Model discrimination was assessed using the area under the receiver operating characteristic curve (AUC/C-statistic), and calibration was evaluated using the Hosmer–Lemeshow test and calibration plots.

Given the observational design, confounding by indication and referral bias were anticipated. To address this, all clinically relevant variables available at the time of referral were included in the model. While residual confounding cannot be excluded, the analysis was designed to estimate adjusted associations rather than causal effects.

The analytical framework prioritizes clinical realism over methodological idealization and was intentionally aligned with available clinical data to preserve validity in real-world settings rather than impose additional assumptions required for causal modeling that may not be justified given the available data structure. More advanced causal inference approaches, such as propensity score methods, were not applied due to the limited number of reliably available retrospective variables. Accordingly, the analysis was designed to estimate adjusted associations rather than causal effects.

All analyses were conducted using Python (version 3.X; Python Software Foundation, Wilmington, DE, USA) within a reproducible computational environment. Standard scientific libraries including pandas, NumPy, statsmodels, and matplotlib were used. Fully reproducible analysis code is provided in Supplementary File S1. All analyses followed prespecified analytic steps. A two-sided *p*-value < 0.05 was considered statistically significant.

### 2.10. Handling of Missing Data

All variables were complete, and no imputation was required.

### 2.11. Ethical Considerations

This study was approved by the Jazan Health Cluster Ethics Committee (Approval No. 25172; Approval Date: 15 December 2025). The study was conducted in accordance with the Declaration of Helsinki (1975, revised in 2013). The requirement for informed consent was waived due to the retrospective nature of the study and the use of anonymized data.

## 3. Results

### 3.1. Study Cohort

**Figure 1:**
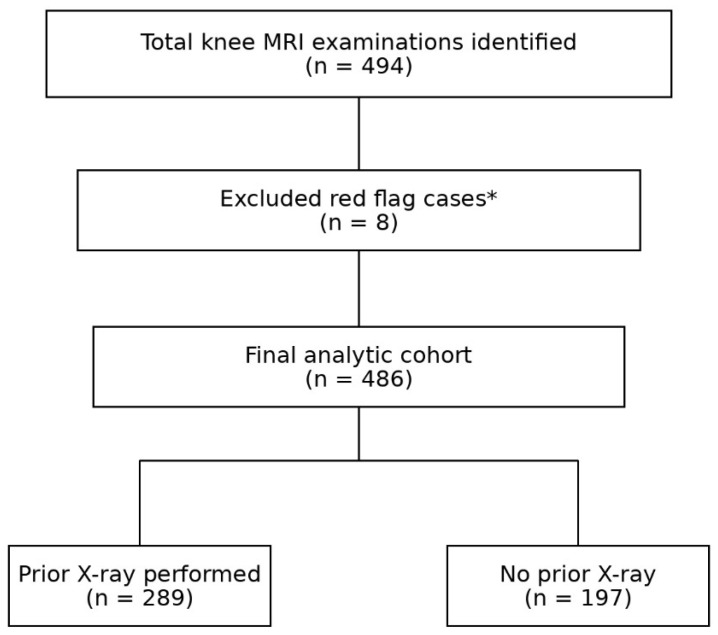
Study flow diagram. A total of 494 knee MRI examinations were identified. Red flag cases (n = 8) were excluded. The final cohort included 486 patients, stratified by prior X-ray status into prior X-ray (n = 289) and no prior X-ray (n = 197). * Red flag cases included suspected serious pathology (e.g., infection, malignancy, or acute fracture).

A total of 494 knee magnetic resonance imaging (MRI) examinations were identified (Figure 1). Red flag cases (n = 8) were excluded, yielding a final analytic cohort of 486 patients. Red flag cases were defined as examinations with suspected serious pathology, including infection, malignancy, or acute fracture.

Among the included patients, 289 (59.5%) underwent MRI following a prior X-ray, while 197 (40.5%) had no prior X-ray.

### 3.2. Baseline Characteristics

Baseline characteristics according to prior X-ray status are presented in Table 1. No formal statistical comparisons were performed for baseline variables, as recommended for descriptive baseline reporting in observational studies; standardized mean differences were reported to describe the magnitude of group imbalance. Clinically relevant MRI findings (as defined in Methods) were observed in 116 patients (40.1%) in the prior X-ray group and 98 patients (49.7%) in the no prior X-ray group, corresponding to an absolute difference of 9.6%.

### 3.3. Association Between Prior X-Ray and MRI Yield

MRI examinations performed following prior X-ray demonstrated a lower frequency of clinically relevant MRI findings compared with examinations performed without prior X-ray.

**Figure 2:**
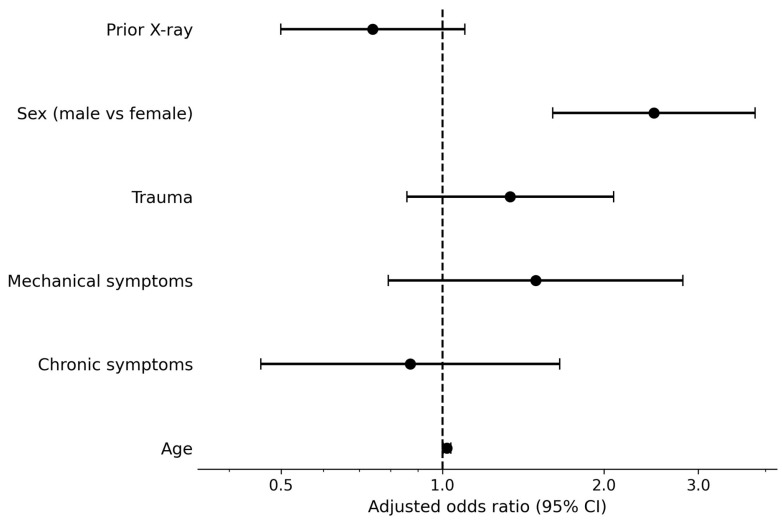
Adjusted associations with clinically relevant MRI findings. The dots represent adjusted odds ratios (aORs), the horizontal lines represent 95% confidence intervals, and the vertical dashed line indicates the null value (OR = 1).

In the multivariable model adjusted for age, sex, trauma status, mechanical symptoms, and symptom duration (Table 2 and Figure 2), prior X-ray showed lower odds of clinically relevant MRI findings, although the association was attenuated after adjustment and no longer statistically significant (adjusted odds ratio, 0.74; 95% confidence interval, 0.50–1.10; *p* = 0.138). Male sex was independently associated with higher odds of clinically relevant MRI findings (adjusted odds ratio, 2.48; 95% confidence interval, 1.61–3.83; *p* < 0.001).

### 3.4. Trauma-Stratified Analysis

Among patients without trauma (n = 316), prior X-ray was associated with lower odds of clinically relevant findings, although the association was not statistically significant after sex-adjusted multivariable modelling (adjusted odds ratio, 0.66; 95% confidence interval, 0.40–1.09; *p* = 0.104). Among trauma patients (n = 170), no significant association was observed (adjusted odds ratio, 0.87; 95% confidence interval, 0.45–1.68; *p* = 0.675) (Table 3).

Formal interaction testing did not show statistically significant effect modification by trauma status (prior X-ray × trauma interaction *p* = 0.317; Table 4).

### 3.5. Sensitivity Analysis

After excluding osteoarthritis cases (n = 432), the direction of association remained similar, although the association was not statistically significant after adjustment (adjusted odds ratio, 0.76; 95% confidence interval, 0.50–1.15; *p* = 0.193) (Table 5).

### 3.6. Model Diagnostics and Performance

All variables were complete for the 486 included patients, and no imputation was required. Assessment of multicollinearity demonstrated no evidence of problematic correlation among model predictors, with all variance inflation factors below commonly accepted thresholds (Supplementary File S2).

The multivariable logistic regression model demonstrated modest discrimination for clinically relevant MRI findings, with an area under the receiver operating characteristic curve (AUC/C-statistic) of 0.647. Calibration assessment did not demonstrate substantial deviation between predicted and observed outcome probabilities (Hosmer–Lemeshow χ^2^ = 11.23, df = 8, *p* = 0.189).

Detailed model performance diagnostics, including discrimination, calibration, the receiver operating characteristic curve, and the calibration plot, are provided in Supplementary Files S2–S5.

## 4. Discussion

MRI diagnostic yield in routine clinical practice may vary according to patient characteristics, clinical context, and referral-related factors. Although patients with prior X-ray demonstrated a lower unadjusted frequency of clinically relevant MRI findings, this association was attenuated after adjustment and was no longer statistically significant. These findings suggest that differences in patient characteristics and referral-related factors may contribute to observed variation in MRI diagnostic yield across imaging pathway groups.

From a healthcare perspective, these findings suggest that diagnostic yield should not be interpreted solely as a reflection of imaging effectiveness. Instead, variation in MRI yield may be associated with differences in imaging pathways and patient selection. In everyday practice, differences in yield may reflect variation in both patient selection and imaging pathways. The relative contribution of these factors cannot be determined from the present observational study. Interpreting MRI yield without considering referral-related factors and patient characteristics may therefore lead to incomplete conclusions regarding imaging performance.

Lower MRI yield may reflect imaging examinations that identify fewer clinically relevant abnormalities; however, the impact of these findings on subsequent clinical management was not evaluated in the present study. In this context, variation in diagnostic yield may reflect differences in how imaging is utilized across healthcare systems. Differences in imaging pathways may therefore have implications for healthcare resource utilization, although outcomes such as cost, waiting times, and access to imaging services were not directly evaluated in the present study. These observations are consistent with broader concerns regarding variation in imaging utilization and the potential overuse of advanced imaging modalities [2,10].

Confounding by indication remains an important consideration. Patients who undergo X-ray before MRI may differ in meaningful ways from those referred directly to MRI, including differences in symptoms, clinical uncertainty, and the likelihood of significant pathology. Consequently, observed variation in MRI yield may reflect differences in patient selection, clinical context, imaging pathways, or other unmeasured factors. The relative contribution of these factors cannot be determined from the present observational study. While residual confounding cannot be ruled out, this is an inherent limitation of real-world retrospective analyses.

These findings are relevant not only to radiology practice but also to the broader healthcare system. Diagnostic yield should be interpreted within the context of patient selection, clinical context, and imaging pathways rather than as a fixed property of the imaging test itself. The findings highlight the importance of considering referral-related factors when evaluating variation in MRI utilization and diagnostic yield. This interpretation is consistent with existing literature emphasizing the importance of appropriate imaging use and the limitations of relying solely on test performance metrics when assessing healthcare utilization and imaging value [1,5].

The subgroup analysis should be interpreted cautiously because formal interaction testing did not demonstrate statistically significant effect modification by trauma status (interaction *p* = 0.317). Although the estimated effect sizes differed between trauma and non-trauma patients, no statistically significant association was observed in either subgroup after adjustment. These findings suggest that the relationship between prior X-ray and MRI diagnostic yield does not differ substantially according to trauma status within the limitations of the present dataset.

Previous work has highlighted the system-level consequences of low-value imaging, including increased healthcare costs and reduced access to imaging services [1,5,11]. The present findings add to this literature by emphasizing that variation in MRI diagnostic yield may be influenced by differences in patient selection, clinical context, and imaging pathways. These considerations may be important when interpreting diagnostic yield as a measure of imaging utilization and healthcare value in routine clinical practice.

This study has several limitations. It was conducted at a single center, and referral patterns may vary across institutions and healthcare settings, potentially limiting generalizability. The outcome was based on radiology report impressions without independent reassessment or interobserver reliability testing. However, this approach reflects how imaging findings are interpreted and used in routine clinical practice and may therefore better capture real-world decision making. Residual confounding remains possible, and causal conclusions cannot be drawn from this observational design. In addition, several potentially relevant variables, including referring specialty, radiographic findings, osteoarthritis severity, prior treatment history, and downstream management outcomes, were not consistently available in structured form and therefore could not be incorporated into the analysis. Radiographic findings themselves were not incorporated into the analysis. Although prior X-ray utilization was evaluated as the primary exposure, radiographic reports were not consistently available in a structured format suitable for reliable retrospective extraction across the study period. Consequently, the study could not assess whether specific radiographic findings influenced subsequent MRI utilization or diagnostic yield. Furthermore, the multivariable model demonstrated only modest discrimination, suggesting that additional unmeasured factors may contribute to variation in MRI diagnostic yield.

## 5. Conclusions

MRI diagnostic yield in routine clinical practice may be influenced by differences in patient selection, clinical context, and imaging pathways. Although patients with prior X-ray demonstrated a lower unadjusted frequency of clinically relevant MRI findings, the association was attenuated after adjustment for sex and other clinical covariates and was no longer statistically significant. These findings highlight the importance of interpreting diagnostic yield within the broader context of healthcare delivery and referral practices. Further studies incorporating additional referral-level variables and downstream clinical outcomes are needed to better understand the factors contributing to variation in MRI diagnostic yield.

## Figures and Tables

**Table 1:** Baseline characteristics according to prior X-ray status.

Variable	No Prior X-Ray (n = 197)	Prior X-Ray (n = 289)	SMD
Age, mean ± SD (years)	37.75 ± 10.13	42.04 ± 12.52	0.37
Sex (male), n (%)	163 (82.7%)	169 (58.5%)	−0.53
Trauma, n (%)	63 (32.0%)	107 (37.0%)	0.11
Mechanical symptoms, n (%)	22 (11.2%)	35 (12.1%)	0.03
Chronic symptoms, n (%)	178 (90.4%)	254 (87.9%)	−0.08
Clinically relevant MRI findings, n (%)	98 (49.7%)	116 (40.1%)	−0.19

SMD = standardized mean difference; values describe the prior X-ray group relative to the no prior X-ray group.

**Table 2:** Multivariable logistic regression model.

Variable	Adjusted Odds Ratio	95% Confidence Interval	*p*-Value
Prior X-ray	0.74	0.50–1.10	0.138
Age (per year)	1.02	1.00–1.04	0.022
Sex (male)	2.48	1.61–3.83	<0.001
Trauma	1.34	0.86–2.09	0.199
Mechanical symptoms	1.49	0.79–2.81	0.215
Chronic symptoms	0.87	0.46–1.65	0.673

**Table 3:** Stratified analysis by trauma status.

Subgroup	N	Adjusted Odds Ratio	95% Confidence Interval	*p*-Value
Non-trauma	316	0.66	0.40–1.09	0.104
Trauma	170	0.87	0.45–1.68	0.675

**Table 4:** Formal interaction testing model.

Subgroup	Odds Ratio	95% Confidence Interval	*p*-Value
Prior X-ray	0.64	0.40–1.04	0.074
Trauma	1.05	0.55–2.01	0.883
Prior X-ray × Trauma	1.50	0.68–3.32	0.317

**Table 5:** Sensitivity analysis excluding osteoarthritis cases.

Analysis	N	Adjusted Odds Ratio	95% Confidence Interval	*p*-Value
Primary analysis	486	0.64	0.40–1.04	0.074
Excluding osteoarthritis cases	432	0.76	0.50–1.15	0.193

## Data Availability

The data supporting the findings of this study are available from the corresponding author upon reasonable request. Due to institutional regulations and patient privacy considerations, the data are not publicly available.

## Supplementary Materials

The following supporting information can be downloaded at https://www.mdpi.com/article/10.3390/healthcare14121628/s1: File S1: Preparation, Statistical Analysis, and Figure Generation; File S2: Multicollinearity Assessment (Variance Inflation Factors); File S3: Model Performance Diagnostics; File S4: ROC Curve; File S5: Calibration Plot.
