# Supplementary material for: Prior X-Ray and Diagnostic Yield of Knee MRI: A Retrospective Study of Imaging Pathways and Healthcare Utilization
Source: Healthcare (Basel). 2026 Jun 9;14(12):1628. doi: 10.3390/healthcare14121628 (PMC13299063; doi:10.3390/healthcare14121628)
Supplement: Supplementary file 1 [file healthcare-14-01628-s001.zip › Supplementary File S1.pdf]

Supplementary File S1

Python Code for Data Preparation, Statistical Analysis, and Figure Generation

The code is provided to facilitate reproducibility of the statistical analyses. Variable derivation procedures for prior X-ray status and clinically relevant MRI findings were completed before statistical modeling according to the definitions described in the Methods section of the manuscript.

Prior X-ray status (prior_xray) was defined as ipsilateral knee radiography performed within 3 months before MRI.

The outcome variable (positive_yield) was derived before statistical analysis according to the predefined classification protocol based on the final MRI report impression.

The original dataset is not publicly available because of patient confidentiality restrictions.

Software environment: Python 3.12, pandas 2.x, NumPy 2.x, statsmodels 0.14.x, scikit-learn 1.x, SciPy 1.x, matplotlib 3.x.

Note. This supplementary file compiles the Python code used to prepare the analysis dataset, reproduce the main statistical analyses, and generate the study figures. The analyzed dataset should be saved in the working directory using the file name "data analysis file.xlsx".

1. Data import and cohort filtering

Code

```
# Supplementary File S1
# Python Code for Data Preparation, Statistical Analysis, and Figure Generation
# Knee MRI utilization study

import pandas as pd
import numpy as np
import statsmodels.api as sm
from statsmodels.stats.outliers_influence import variance_inflation_factor
from sklearn.metrics import roc_auc_score, roc_curve
from sklearn.calibration import calibration_curve
from scipy import stats
import matplotlib.pyplot as plt

# The analyzed dataset should be named exactly as follows:
# data analysis file.xlsx

df = pd.read_excel("data analysis file.xlsx")

# Exclude red flag cases
df = df[df["red_flag"] == 0].copy()

# Confirm final analytic cohort
print("Final analytic cohort:")
print(df.shape)
```

2. Descriptive statistics and Table 1

Code

```
print("\nPrior X-ray distribution:")
print(df["prior_xray"].value_counts())
print(df["prior_xray"].value_counts(normalize=True))

print("\nClinically relevant MRI findings by prior X-ray status:")
print(df.groupby("prior_xray")["positive_yield"].mean())

def standardized_mean_difference_continuous(x0, x1):
    mean0 = x0.mean()
    mean1 = x1.mean()
    sd0 = x0.std(ddof=1)
    sd1 = x1.std(ddof=1)
    n0 = len(x0)
    n1 = len(x1)
    pooled_sd = np.sqrt(((n0 - 1) * sd0**2 + (n1 - 1) * sd1**2) / (n0 + n1 - 2))
    return (mean1 - mean0) / pooled_sd

def standardized_mean_difference_binary(p0, p1):
    pooled_p = (p0 + p1) / 2
    return (p1 - p0) / np.sqrt(pooled_p * (1 - pooled_p))

table1_rows = []

variables = [
    ("age", "Age, mean +/- SD (years)", "continuous"),
    ("sex_binary", "Sex (male), n (%)", "binary"),
    ("trauma", "Trauma, n (%)", "binary"),
    ("mechanical_symptoms", "Mechanical symptoms, n (%)", "binary"),
    ("symptom_duration", "Chronic symptoms, n (%)", "binary"),
    ("positive_yield", "Clinically relevant MRI findings, n (%)", "binary")
]

for var, label, var_type in variables:
    no_xray = df[df["prior_xray"] == 0]
    yes_xray = df[df["prior_xray"] == 1]

    if var_type == "continuous":
        no_value = f"{no_xray[var].mean():.2f} +/- {no_xray[var].std(ddof=1):.2f}"
        yes_value = f"{yes_xray[var].mean():.2f} +/- {yes_xray[var].std(ddof=1):.2f}"
        smd = standardized_mean_difference_continuous(no_xray[var], yes_xray[var])
    else:
        no_n = no_xray[var].sum()
        yes_n = yes_xray[var].sum()
        no_p = no_n / len(no_xray)
        yes_p = yes_n / len(yes_xray)
        no_value = f"{int(no_n)} ({no_p * 100:.1f}%)"
        yes_value = f"{int(yes_n)} ({yes_p * 100:.1f}%)"
        smd = standardized_mean_difference_binary(no_p, yes_p)

    table1_rows.append({
        "Variable": label,
        "No prior X-ray": no_value,
        "Prior X-ray": yes_value,
        "SMD": round(smd, 2)
    })

table1 = pd.DataFrame(table1_rows)
print("\nTable 1:")
```

```
print(table1)
```

3. Unadjusted logistic regression

Code

```
X = df[["prior_xray"]]
y = df["positive_yield"]
X = sm.add_constant(X)

model_unadj = sm.Logit(y, X).fit(disp=0)

print("\nUnadjusted logistic regression:")
print(model_unadj.summary())

params = model_unadj.params
conf = model_unadj.conf_int()

or_unadj = pd.DataFrame({
    "OR": np.exp(params),
    "Lower CI": np.exp(conf[0]),
    "Upper CI": np.exp(conf[1]),
    "p-value": model_unadj.pvalues
})

print("\nUnadjusted OR table:")
print(or_unadj)
```

4. Multivariable logistic regression including sex

Code

```
predictors = [
    "prior_xray",
    "age",
    "sex_binary",
    "trauma",
    "mechanical_symptoms",
    "symptom_duration"
]

X = df[predictors]
y = df["positive_yield"]
X = sm.add_constant(X)

model = sm.Logit(y, X).fit(disp=0)

print("\nMultivariable logistic regression:")
print(model.summary())

params = model.params
conf = model.conf_int()

or_table = pd.DataFrame({
    "OR": np.exp(params),
    "Lower CI": np.exp(conf[0]),
    "Upper CI": np.exp(conf[1]),
    "p-value": model.pvalues
```

```
})

print("\nAdjusted OR table:")
print(or_table)
```

5. Formal interaction testing by trauma status

Code

```
df["priorxray_trauma"] = df["prior_xray"] * df["trauma"]

interaction_predictors = [
    "prior_xray",
    "age",
    "sex_binary",
    "trauma",
    "mechanical_symptoms",
    "symptom_duration",
    "priorxray_trauma"
]

X_int = df[interaction_predictors]
y_int = df["positive_yield"]
X_int = sm.add_constant(X_int)

interaction_model = sm.Logit(y_int, X_int).fit(disp=0)

print("\nInteraction model:")
print(interaction_model.summary())

params = interaction_model.params
conf = interaction_model.conf_int()

interaction_or_table = pd.DataFrame({
    "OR": np.exp(params),
    "Lower CI": np.exp(conf[0]),
    "Upper CI": np.exp(conf[1]),
    "p-value": interaction_model.pvalues
})

print("\nInteraction OR table:")
print(interaction_or_table)

print("\nInteraction p-value for prior X-ray x trauma:")
print(interaction_model.pvalues["priorxray_trauma"])
```

6. Stratified analysis by trauma status

Code

```
# Non-trauma subgroup

df_non_trauma = df[df["trauma"] == 0].copy()

X_nt = df_non_trauma[[
    "prior_xray",
    "age",
```

```
    "sex_binary",
    "mechanical_symptoms",
    "symptom_duration"
]]

y_nt = df_non_trauma["positive_yield"]
X_nt = sm.add_constant(X_nt)

model_nt = sm.Logit(y_nt, X_nt).fit(disp=0)

print("\nNon-trauma subgroup model:")
print(model_nt.summary())

params_nt = model_nt.params
conf_nt = model_nt.conf_int()

or_nt = pd.DataFrame({
    "OR": np.exp(params_nt),
    "Lower CI": np.exp(conf_nt[0]),
    "Upper CI": np.exp(conf_nt[1]),
    "p-value": model_nt.pvalues
})

print("\nNon-trauma subgroup OR table:")
print(or_nt)

# Trauma subgroup

df_trauma = df[df["trauma"] == 1].copy()

X_t = df_trauma[[
    "prior_xray",
    "age",
    "sex_binary",
    "mechanical_symptoms",
    "symptom_duration"
]]

y_t = df_trauma["positive_yield"]
X_t = sm.add_constant(X_t)

model_t = sm.Logit(y_t, X_t).fit(disp=0)

print("\nTrauma subgroup model:")
print(model_t.summary())

params_t = model_t.params
conf_t = model_t.conf_int()

or_t = pd.DataFrame({
    "OR": np.exp(params_t),
    "Lower CI": np.exp(conf_t[0]),
    "Upper CI": np.exp(conf_t[1]),
    "p-value": model_t.pvalues
})

print("\nTrauma subgroup OR table:")
```

```
print(or_t)
```

7. Sensitivity analysis excluding osteoarthritis

Code

```
df_no_oa = df[df["osteoarthritis"] == 0].copy()

X_sens = df_no_oa[[
    "prior_xray",
    "age",
    "sex_binary",
    "trauma",
    "mechanical_symptoms",
    "symptom_duration"
]]

y_sens = df_no_oa["positive_yield"]
X_sens = sm.add_constant(X_sens)

model_sens = sm.Logit(y_sens, X_sens).fit(disp=0)

print("\nSensitivity analysis excluding osteoarthritis:")
print(model_sens.summary())

params_sens = model_sens.params
conf_sens = model_sens.conf_int()

or_sens = pd.DataFrame({
    "OR": np.exp(params_sens),
    "Lower CI": np.exp(conf_sens[0]),
    "Upper CI": np.exp(conf_sens[1]),
    "p-value": model_sens.pvalues
})

print("\nSensitivity OR table:")
print(or_sens)
```

8. Multicollinearity diagnostics

Code

```
X_vif = df[[
    "prior_xray",
    "age",
    "sex_binary",
    "trauma",
    "mechanical_symptoms",
    "symptom_duration"
]]

X_vif = sm.add_constant(X_vif)

vif = pd.DataFrame()
vif["Variable"] = X_vif.columns
vif["VIF"] = [
    variance_inflation_factor(X_vif.values, i)
    for i in range(X_vif.shape[1])
```

```
]

print("\nVariance inflation factors:")
print(vif)
```

9. Model performance: discrimination and calibration

Code

```
pred_prob = model.predict(X)
auc = roc_auc_score(y, pred_prob)

print("\nAUC / C-statistic:")
print(round(auc, 3))

# Hosmer-Lemeshow calibration test

hl_df = pd.DataFrame({
    "observed": y,
    "predicted": pred_prob
})

hl_df["decile"] = pd.qcut(
    hl_df["predicted"],
    10,
    duplicates="drop"
)

hl_table = hl_df.groupby("decile", observed=False).agg(
    observed_events=("observed", "sum"),
    expected_events=("predicted", "sum"),
    n=("observed", "size")
)

hl_table["observed_nonevents"] = hl_table["n"] - hl_table["observed_events"]
hl_table["expected_nonevents"] = hl_table["n"] - hl_table["expected_events"]

hl_stat = (
    ((hl_table["observed_events"] - hl_table["expected_events"]) ** 2)
    / (hl_table["expected_events"] + 1e-9)
    +
    ((hl_table["observed_nonevents"] - hl_table["expected_nonevents"]) ** 2)
    / (hl_table["expected_nonevents"] + 1e-9)
).sum()

hl_degrees_freedom = len(hl_table) - 2
hl_p = 1 - stats.chi2.cdf(hl_stat, hl_degrees_freedom)

print("\nHosmer-Lemeshow calibration test:")
print("Chi-square:", round(hl_stat, 2))
print("df:", hl_degrees_freedom)
print("p-value:", round(hl_p, 3))
```

10. Export statistical tables

Code

```
table1.to_csv("Table1_Baseline_Characteristics.csv", index=False)
```

```
or_table.to_csv("Table2_Multivariable_Logistic_Regression.csv")
interaction_or_table.to_csv("Table4_Interaction_Model.csv")
or_nt.to_csv("NonTrauma_Subgroup_Model.csv")
or_t.to_csv("Trauma_Subgroup_Model.csv")
or_sens.to_csv("Sensitivity_Analysis_Excluding_OA.csv")
vif.to_csv("Supplementary_Table_S1_VIF.csv", index=False)
hl_table.to_csv("Calibration_Hosmer_Lemeshow_Table.csv")
```

11. Figure 1 flowchart generation

Code

```
from PIL import Image, ImageDraw, ImageFont

W, H = 1000, 700
img = Image.new("RGB", (W, H), "white")
draw = ImageDraw.Draw(img)

try:
    font = ImageFont.truetype("DejaVuSans.ttf", 28)
except:
    font = ImageFont.load_default()

def centered_multiline(draw, box, text, font):
    x1, y1, x2, y2 = box
    lines = text.split("\n")
    line_heights = []
    line_widths = []

    for line in lines:
        b = draw.textbbox((0, 0), line, font=font)
        line_widths.append(b[2] - b[0])
        line_heights.append(b[3] - b[1])

    total_h = sum(line_heights) + (len(lines) - 1) * 6
    y = y1 + (y2 - y1 - total_h) / 2

    for line, lw, lh in zip(lines, line_widths, line_heights):
        x = x1 + (x2 - x1 - lw) / 2
        draw.text((x, y), line, fill="black", font=font)
        y += lh + 6

def box(x1, y1, x2, y2, text):
    draw.rectangle([x1, y1, x2, y2], outline="black", width=3)
    centered_multiline(draw, (x1, y1, x2, y2), text, font)

def line(x1, y1, x2, y2):
    draw.line([x1, y1, x2, y2], fill="black", width=3)

box(300, 40, 700, 120, "Total knee MRI examinations\n(n = 494)")
box(300, 180, 700, 260, "Excluded red flag cases\n(n = 8)")
box(300, 320, 700, 400, "Final analytic cohort\n(n = 486)")
box(90, 520, 430, 610, "Prior X-ray\n(n = 289)")
box(570, 520, 910, 610, "No prior X-ray\n(n = 197)")

line(500, 120, 500, 180)
line(500, 260, 500, 320)
line(500, 400, 500, 445)
```

```
line(500, 445, 260, 520)
line(500, 445, 740, 520)

img.save("Figure1_Flowchart.tiff", dpi=(300, 300))
img.save("Figure1_Flowchart.png", dpi=(300, 300))
```

12. Figure 2 forest plot generation

Code

```
label_map = {
    "prior_xray": "Prior X-ray",
    "sex_binary": "Sex (male vs female)",
    "trauma": "Trauma",
    "mechanical_symptoms": "Mechanical symptoms",
    "symptom_duration": "Chronic symptoms",
    "age": "Age"
}

params_plot = model.params.drop("const")
conf_plot = model.conf_int().drop("const")

plot_df = pd.DataFrame({
    "Variable": params_plot.index,
    "Label": [label_map[v] for v in params_plot.index],
    "OR": np.exp(params_plot.values),
    "Lower": np.exp(conf_plot[0].values),
    "Upper": np.exp(conf_plot[1].values)
})

order = [
    "prior_xray",
    "sex_binary",
    "trauma",
    "mechanical_symptoms",
    "symptom_duration",
    "age"
]

plot_df = plot_df.set_index("Variable").loc[order].reset_index()
plot_df = plot_df.iloc[::-1].reset_index(drop=True)

y_pos = np.arange(len(plot_df))

plt.figure(figsize=(8.0, 5.4))

plt.errorbar(
    plot_df["OR"],
    y_pos,
    xerr=[
        plot_df["OR"] - plot_df["Lower"],
        plot_df["Upper"] - plot_df["OR"]
    ],
    fmt="o",
    color="black",
    ecolor="black",
    elinewidth=1.8,
    capsize=4,
    markersize=7
```

```
)

plt.yticks(y_pos, plot_df["Label"], fontsize=12)
plt.axvline(x=1, color="black", linestyle="--", linewidth=1.5)
plt.xlabel("Adjusted odds ratio (95% CI)", fontsize=12)
plt.xscale("log")
plt.xlim(0.35, 4.2)
plt.xticks([0.5, 1.0, 2.0, 3.0], ["0.5", "1.0", "2.0", "3.0"], fontsize=11)

ax = plt.gca()
ax.spines["top"].set_visible(False)
ax.spines["right"].set_visible(False)
ax.spines["left"].set_visible(False)
ax.tick_params(axis="y", length=0)
ax.grid(False)

plt.tight_layout()
plt.savefig("Figure2_ForestPlot.tiff", dpi=300, bbox_inches="tight")
plt.savefig("Figure2_ForestPlot.png", dpi=300, bbox_inches="tight")
plt.close()
```

13. Figure 3 calibration plot generation

Code

```
prob_true, prob_pred = calibration_curve(
    y,
    pred_prob,
    n_bins=10,
    strategy="quantile"
)

plt.figure(figsize=(5, 5))
plt.plot(prob_pred, prob_true, marker="o", color="black")
plt.plot([0, 1], [0, 1], linestyle="--", color="black")
plt.xlabel("Predicted probability")
plt.ylabel("Observed probability")
plt.title("Calibration Plot")
plt.tight_layout()
plt.savefig("Figure3_CalibrationPlot.tiff", dpi=300, bbox_inches="tight")
plt.savefig("Figure3_CalibrationPlot.png", dpi=300, bbox_inches="tight")
plt.close()
```

14. Supplementary Figure S1 ROC curve generation

Code

```
fpr, tpr, thresholds = roc_curve(y, pred_prob)

plt.figure(figsize=(5, 5))
plt.plot(fpr, tpr, color="black", label=f"AUC = {auc:.3f}")
plt.plot([0, 1], [0, 1], linestyle="--", color="black")
plt.xlabel("False positive rate")
plt.ylabel("True positive rate")
plt.title("ROC Curve")
plt.legend(loc="lower right")
plt.tight_layout()
plt.savefig("Supplementary_Figure_S1_ROC.tiff", dpi=300, bbox_inches="tight")
plt.savefig("Supplementary_Figure_S1_ROC.png", dpi=300, bbox_inches="tight")
plt.close()
```
