# Supplementary material for: Prior X-Ray and Diagnostic Yield of Knee MRI: A Retrospective Study of Imaging Pathways and Healthcare Utilization
Source: Healthcare (Basel). 2026 Jun 9;14(12):1628. doi: 10.3390/healthcare14121628 (PMC13299063; doi:10.3390/healthcare14121628)
Supplement: Supplementary file 1 [file healthcare-14-01628-s001.zip › Supplementary File S2.pdf]

Supplementary File S2: Multicollinearity Assessment (Variance Inflation Factors)

Variable	VIF
Prior X-ray	1.17
Age	1.11
Sex	1.09
Trauma	1.32
Mechanical symptoms	1.06
Chronic symptoms	1.08

Variance inflation factor (VIF) analysis for variables included in the primary multivariable logistic regression model. VIF values were calculated to assess potential multicollinearity among predictors. All values were substantially below commonly accepted thresholds for concern, indicating no evidence of meaningful multicollinearity and supporting the stability and interpretability of the adjusted regression estimates.
