# Supplementary material for: Prior X-Ray and Diagnostic Yield of Knee MRI: A Retrospective Study of Imaging Pathways and Healthcare Utilization
Source: Healthcare (Basel). 2026 Jun 9;14(12):1628. doi: 10.3390/healthcare14121628 (PMC13299063; doi:10.3390/healthcare14121628)
Supplement: Supplementary file 1 [file healthcare-14-01628-s001.zip › Supplementary File S3.pdf]

Supplementary File S3: Model Performance Diagnostics

Metric	Value
AUC / C-statistic	0.647
Hosmer–Lemeshow χ^2	11.23
Degrees of freedom	8
Hosmer–Lemeshow p-value	0.189

Performance diagnostics for the primary multivariable logistic regression model evaluating the association between prior X-ray and clinically relevant MRI findings. Model discrimination was assessed using the area under the receiver operating characteristic curve (AUC/C-statistic). Calibration was evaluated using the Hosmer–Lemeshow goodness-of-fit test. The observed AUC indicates modest discriminative ability, while the non-significant Hosmer–Lemeshow test suggests no evidence of substantial departure between predicted and observed outcome probabilities.
