# Supplementary figures and images for: Prior X-Ray and Diagnostic Yield of Knee MRI: A Retrospective Study of Imaging Pathways and Healthcare Utilization
Source: Healthcare (Basel). 2026 Jun 9;14(12):1628. doi: 10.3390/healthcare14121628 (PMC13299063; doi:10.3390/healthcare14121628)

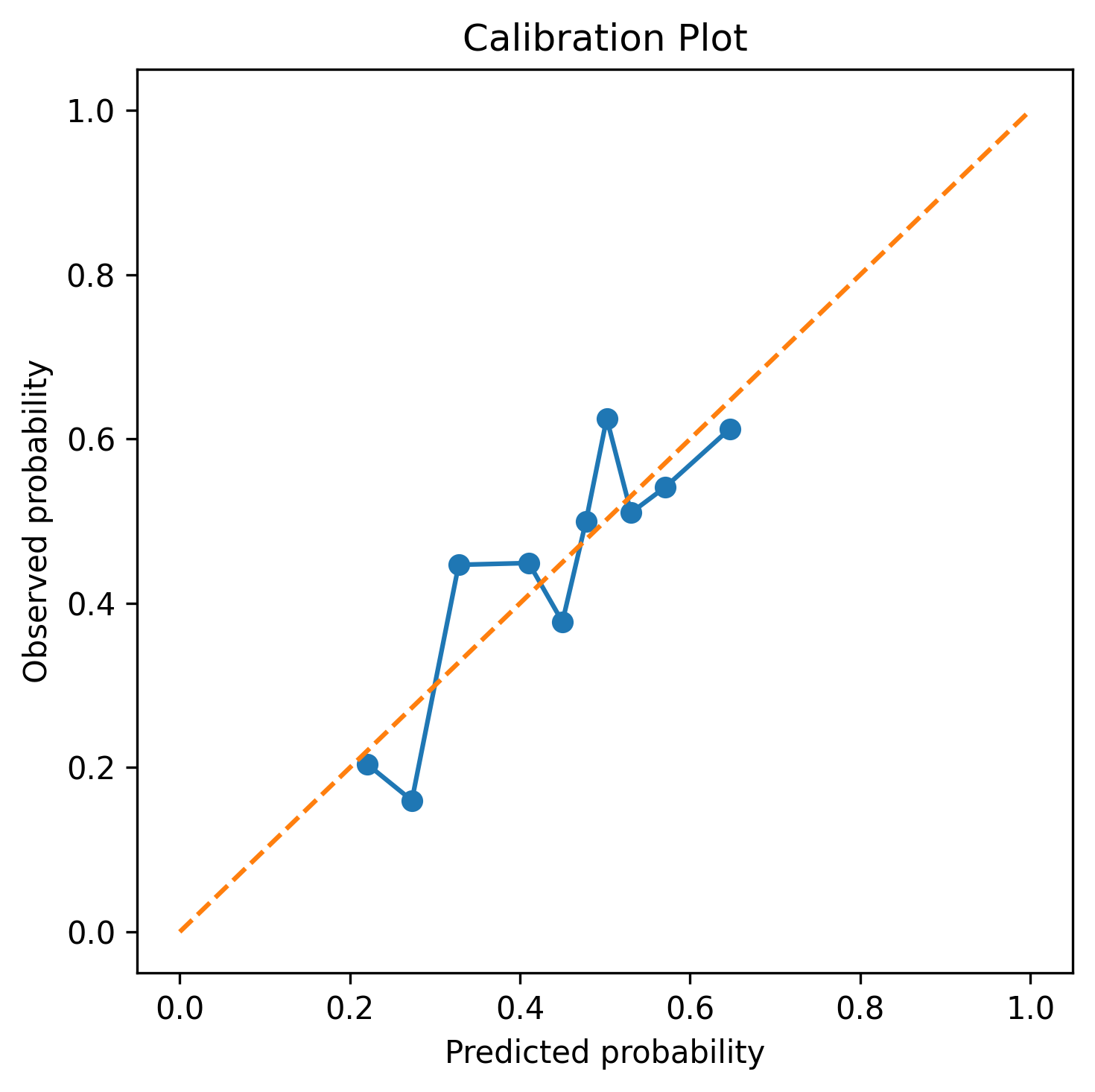

Supplement: Supplementary file 1 [file healthcare-14-01628-s001.zip › Supplementary File S5 – Calibration Plot.tiff]

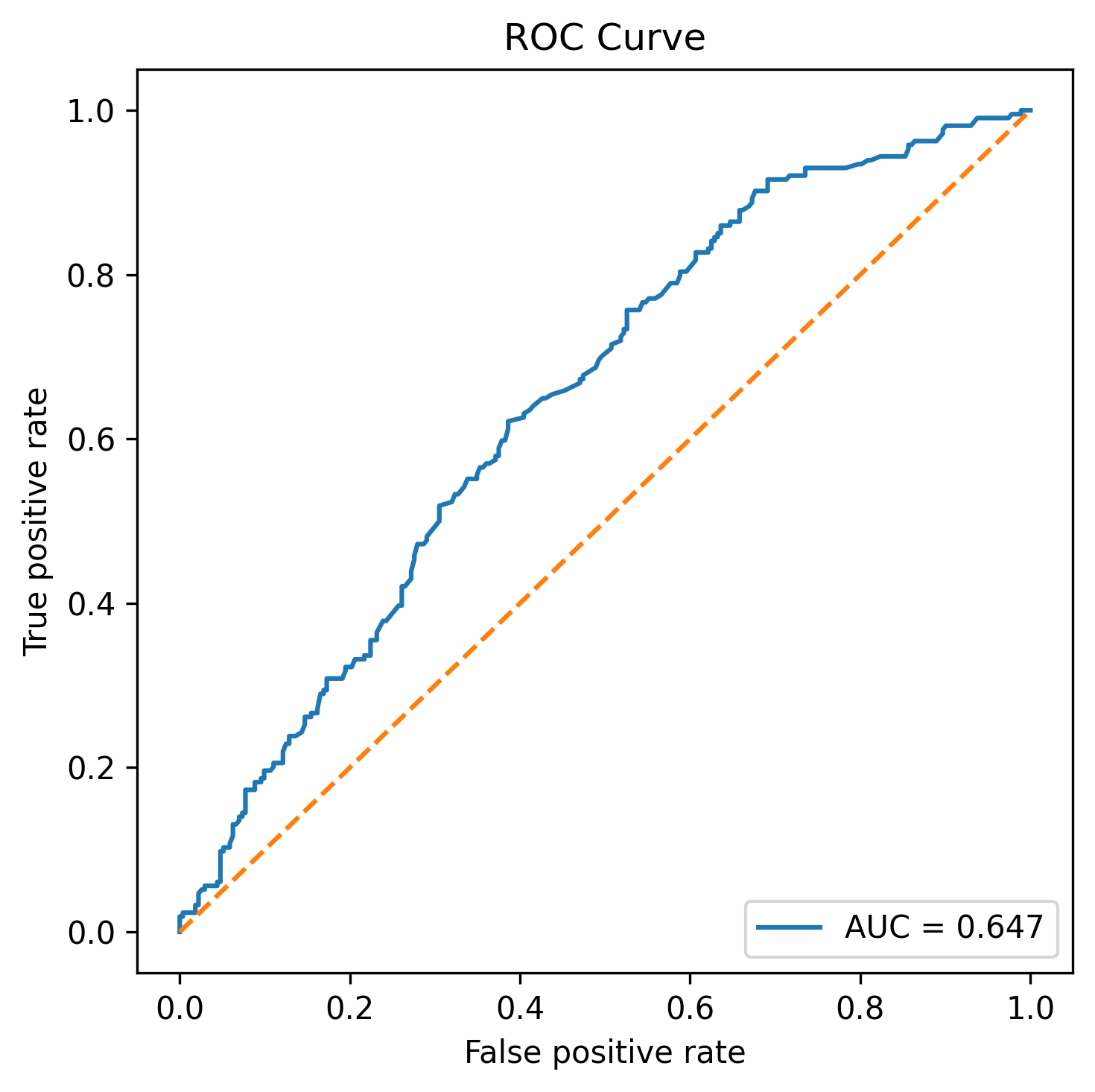

Supplement: Supplementary file 1 [file healthcare-14-01628-s001.zip › Supplementary File S4 – ROC Curve.tiff]
